# 多发性骨髓瘤微小残留病检测与应用进展

**DOI:** 10.3760/cma.j.cn121090-20230728-00036

**Published:** 2024-02

**Authors:** 慧星 周, 文明 陈

**Affiliations:** 首都医科大学附属北京朝阳医院血液科，北京市多发性骨髓瘤医疗研究中心，北京 100020 Department of Hematology, Beijing Chaoyang Hospital, Capital Medical University, Multiple Myeloma Research Center of Beijing, Beijing 100020, China

## Abstract

多发性骨髓瘤的治疗药物在近20年来快速迭代，患者缓解率和缓解深度不断提高，传统的疾病疗效监测方法不能满足新药时代的临床需求。微小残留病（MRD）是一种更为敏感的用于评估疾病缓解深度的检测手段，多项临床试验数据和荟萃分析显示，相比较传统的完全缓解，MRD阴性与更好的预后相关，MRD驱动治疗也成为研究焦点。目前MRD评估涉及三个维度：基于骨髓克隆性浆细胞的MRD检测，基于局灶病变残留代谢活性的MRD检测，基于外周血的MRD检测。各个MRD的评估方法互为补充。本文旨在系统性介绍多发性骨髓瘤MRD检测方法与应用进展，为临床医师更好运用相关技术提供参考。

## Abstract (en)

With the rapid iteration of multiple myeloma therapeutics over the last two decades, as well as increasing remission rates and depth of remission in patients, traditional methods for monitoring disease response are insufficient to meet the clinical needs of new drugs. Minimal residual disease (MRD) is a more sensitive test for determining the depth of response, and data from multiple clinical trials and meta-analyses show that a negative MRD correlates with a better prognosis than a traditional complete response. MM is at the forefront of MRD evaluation and treatment. MRD detection methods have been continuously updated. The current MRD assessment has three dimensions: bone marrow-based MRD testing, MRD testing based on images of residual metabolic of focal lesions, and peripheral blood-based MRD testing. The various MRD assessment methods complement one another. The goal of this article is to discuss the currently used MRD assays, the progress, and challenges of MRD in MM, and to provide a reference for clinicians to better use the techniques.

近20年来，越来越多的新药获批治疗多发性骨髓瘤（MM），新物的应用使MM的缓解率和缓解深度得到显著提高，更多患者获得微小残留病（MRD）阴性[1]–[2]。MM的MRD指MM经过治疗达到血液学完全缓解后体内残存的通过形态学等传统方法无法检测到的微量肿瘤细胞。鉴于MM的特殊性即克隆性浆细胞分泌单克隆免疫球蛋白、浆细胞在骨髓灶性生长且可能伴有浆细胞瘤等状态，MM-MRD的检测不仅要检测骨髓克隆性浆细胞，还需要同时检测局灶病变部位的残留代谢活性。此外，还可以进行基于外周血的MRD检测。

基于以上三个方面，MM-MRD检测手段包括：骨髓克隆性浆细胞检测：多参数流式细胞术（MFC）、二代测序（next generation sequencing，NGS）等；影像学检测病灶部位代谢活性：PET-CT、全身弥散核磁（diffusion weighted imaging-magnetic resonance，DWI-MRI）等；检测外周血中克隆性浆细胞分泌的M蛋白或片段：血清重轻链（Hevylite^®^）、基于质谱（mass spectrometry，MS）技术的M蛋白分析等。由于骨髓瘤细胞具有灶性分布的特征，并且骨髓克隆性浆细胞数量或者比例与外周血M蛋白不成正比，因此没有一种MRD评估能够全面反映疾病的缓解状况，本文将介绍MM-MRD目前的检测方法及临床应用，有助于临床医师更好评估MM治疗效果、预测预后，协助临床医师进行治疗决策。

一、基于骨髓的MM-MRD检测

1. MFC：MFC利用不同荧光标记的多种抗体组合，监测细胞表面或胞内抗原的表达状况，进而对细胞的谱系、分化程度、表型异常与否进行分析判断，是一种高通量、高敏感检测技术。

欧洲流式学会（EuroFlow）建立了2管8色抗体组合的NGF-MRD检测方法。抗体组合分别为：第1管为胞膜抗体，包括CD117 APC、CD19 PC7、CD138 BV421、CD56 PE、CD45 PerCP-Cy5.5、CD81 APC-C750、CD38 FITC和CD27 BV510；第2管为胞质轻链抗体，包括CD19 PC7、CD138 BV421、CD56PE、CD45 PerCP-Cy5.5、CD38 FITC、CD27 BV510、κ APC和λ APC-C750，该荧光组合发射光谱重叠程度低，敏感性最高。EuroFlow的NGF检测MM-MRD需要采集10^7^个细胞，检出限（limit of detection，LOD）为可检测100万个细胞中的2个异常浆细胞，定量限（limit of quantitation，LOQ）为<5×10^−6^，敏感性为10^−5^～10^−6^ [3]–[4]。本方法具有标准操作流程和自动浆细胞设门系统，可以对样本进行自动分析，减少实验室之间的差异，提高检测速度。

纪念斯隆凯特琳癌症中心（Memorial Sloan Kettering Cancer Center，MSKCC）开发了10色单管多参数流式检测MRD（MSKCC单管法），需采集300万个细胞，LOD为可检测100万个细胞中的6个异常浆细胞，敏感性为10^−5^，略低于EuroFlow方法。抗体组合包括CD117 PC5.5、CD19 PC7、CD138APC、CD56 APC-R700、CD45 APC-H7、CD81 Pacific Blue、CD38 BV510、CD27 BV605、κ FITC和λ PE[5]。单管方法相较EuroFlow操作流程简化，成本降低。

NGF MRD优势在于其广泛的适用性、无需基线样本作参照，并且检测耗时短（3～4 h）。缺点在于需要新鲜骨髓标本（细胞活力>85％），样本接收后快速处理（样本采集后的24 h内，不能晚于48 h）。稀释的骨髓样本可影响检测结果。使用“非推荐”的抗体组合、样本处理延迟、检测过程无法获取足够细胞事件等情况会导致检测阈值降低。

2. NGS检测MRD：MRD的分子学方法主要通过检测免疫球蛋白重链或者轻链V（D）J基因重排实现，主要技术手段为等位基因特异性寡核苷酸聚合酶链反应（ASO-PCR）和NGS方法[3],[6]–[7]。ASO-PCR通过检测患者免疫球蛋白重链或轻链可变区中的CDR3重排的克隆性评估MRD，敏感性为10^−5^～10^−6^，需患者基线的特异性重排序列作为参照，临床适用率约70％ [8]。由于ASO-PCR操作繁琐[9]，逐渐被NGS取代。

NGS-MRD是基于ClonoSeq^®^平台的高通量测序方法，在2018年获得美国FDA批准用于MM MRD检测，通过检测肿瘤特异性免疫球蛋白重链可变区V（D）J重排序列，敏感性可达10^−6^。V（D）J重排发生于B细胞抗原依赖体细胞高频突变和类别转换重组前阶段，当B细胞分化为成熟浆细胞后，每一株浆细胞均有其特有的IGH V（D）J可变区重排序列，可以作为浆细胞克隆性的标志。初诊患者NGS检出率为90％～95％，临床适用性好[10]–[11]。另外，Euroclonality-NGS工作组[12]–[14]和LymphoTrack^®^平台[15]–[16] 也获得了质量控制研究的验证。需要强调的是，基于NGS的基因突变或者染色体易位不能作为MRD监测指标。

NGS的优势在于可以利用冻存的样本进行检测，最低300万个细胞的DNA可以满足检测需求；缺点在于NGS-MRD需要患者基线数据作为对照，否则结果无法正确判读。大量的体细胞突变会干扰引物退火和扩增的能力，导致假阴性结果。与NGF类似，由于MM灶性分布的特点，样本采集无法获得足够浆细胞将影响结果。

CASSIOPEIA试验中报道了NGS和NGF两种MRD检测方法一致性为83.5％（<10^−5^水平）[17]。在FORTE研究中，NGF和NGS在完全缓解（CR）及以上疗效的患者中一致性为86％[18]。NGF和NGS均能达到10^−6^检测水平，由于骨髓瘤细胞灶性分布的特点，两种检测方法都存在因血液稀释导致假阴性结果的可能，因而在抽吸MRD骨髓样本时，需强调使用第一次抽吸的骨髓液进行MRD检测。

二、影像学MRD

MM不仅存在溶骨性病变，还表现为髓外病变（extramedullary disease，EMD）[19]，骨旁EMD发生率7％～34％，软组织EMD发生率1％～4％[20]。对复发/难治患者和EMD患者，仅评估骨髓MRD会导致评估偏倚。因此，影像学评估对于局灶病变的评估至关重要[21]。

1. PET-CT：^18^F-氟脱氧葡萄糖（FDG）是PET-CT 最常用的影像学示踪剂，可以同时检测骨病和EMD部位局部代谢情况[22]。2016 IMWG缓解标准明确提出了MM“影像学 MRD”的概念[23]，即骨髓MRD阴性，原有病灶部位标准摄取值（SUV）低于纵隔血池，或低于周围正常组织的SUV。

^18^F-FDG PET-CT对骨病及EMD的评估是对NGF或者NGS-MRD的补充，治疗后PET-CT阴性与良好的无进展生存（PFS）和总生存（OS）相关。但是由于浆细胞己糖激酶水平较低，导致FDG摄取减少，约10％的患者可能会出现假阴性结果[24]。另外，^18^F-FDG PET-CT无法准确区分炎症性疾病，导致出现假阳性结果。该缺陷可以通过使用不依赖己糖激酶的PET示踪剂来解决。^11^C-甲硫氨酸可作为替代FDG的造影剂之一，其摄取与蛋白质合成相关（在MM细胞中非常活跃），检测局灶性病变方面比FDG更敏感[25]。

一种新型靶向成像技术也用于MM影像评估。^89^Zr标记达雷妥尤单克隆抗体（^89^Zr-DFO-Daratumumab）是一种新型免疫PET示踪剂，靶向结合骨髓瘤细胞上的CD38并通过PET 扫描仪显示骨髓瘤病灶，实现MM病灶的可视化[26]。目前正在开展Ⅱ期临床研究（NCT04814615）评估靶向成像技术的临床应用价值。

2. DWI-MRI：MRI检测骨病变部位浆细胞浸润的灵敏度更高，被广泛用于 MM 的诊断。对治疗后的病变监测方面，^18^F-FDG PET-CT有助于显示早期治疗反应，而MRI反应通常延迟。与^18^F-FDG PET-CT相比，MRI在鉴别骨重塑和活性疾病方面特异性较低。因此MRI对残留病灶检测并且不如^18^F-FDG PET-CT[27]。DWI-MRI能够反映病变部位代谢，在治疗后局灶性病变方面的敏感度与PET-CT相似 [28]–[29]，有助于避免X线对患者的危害，价格便宜；与骨髓NGF-MRD的预后价值相当[30]。

FORTE试验比较了PET-CT与骨髓MRD对预后的影响。结果显示，在10^−5^水平，PET-CT与NGS-MRD和NGF-MRD具有较好的一致性，分别为84％和93％。但是在局灶病变的评估方面存在33％～37％差异[31]。因此，PET-CT MRD与骨髓MRD是互补关系。

三、基于外周血的MRD检测

基于外周血的MRD检测相对无创、便捷，能够在一定程度上解决MM的空间异质性、浆细胞灶性分布及血液稀释导致骨髓MRD假阴性的问题，因此，外周血MRD成为一种新兴的监测手段，包括：Hevylite^®^及MS技术检测血清M蛋白；外周血循环浆细胞的检测。但是，外周血MRD与骨髓MRD的一致性还有待更多研究验证。

1. Hevylite^®^：Hevylite^®^能够对每种免疫球蛋白（Ig）类别的不同重链/轻链组合进行定量分析，敏感性显著高于传统蛋白电泳和免疫固定电泳，检测水平可达到0.01～0.02 g/L，特别是能够克服单克隆IgA蛋白电泳时向β区迁移的导致IgA型M蛋白检测不准确的局限性。另外，Hevylite^®^对非受累免疫球蛋白的检测有助于动态监测免疫逃逸和免疫重建[23]。Hevylite^®^ MRD检测的敏感性与MFC检测10^−4^水平相当[32]。

2. MS技术：MS检测血清M蛋白较传统血清蛋白电泳及免疫固定电泳更敏感，主要有两种方法：克隆特征肽（clonotypic peptide）分析和完整免疫球蛋白轻链（intact immunoglobulin light chain）分析，能够定量和定性分析血清M蛋白，并可以区分血清中单抗药物（如达雷妥尤单抗）与单克隆免疫球蛋白成分。通过免疫球蛋白富集能够进一步提高MS检测敏感性。

克隆特征肽法最敏感，可达0.001 g/L，该方法需要基线M蛋白互补决定区序列（complementarity-determining regions，CDR）信息。IFM 2009研究显示克隆特征肽法MS与NGS-MRD一致性为78％[33]。然而该方法操作复杂，耗时长，并且需要针对性设计CDR特征肽，临床适用性低。

完整免疫球蛋白轻链分析MS更便捷、实用，也称为单克隆免疫球蛋白快速精确质谱检测（monoclonal Ig rapid accurate mass measurements，miRAMM）[34]，定量限为0.05 g/L，检测限为0.01 g/L，包括两种检测方法：基质辅助激光解吸电离飞行时间质谱（matrix-assisted laser desorption/ionization time-of-flight mass spectrometry，MALDI-TOF MS）和定量免疫共沉淀质谱（quantitative immunoprecipitation mass spectrometry，QIP-MS）。MALDI-TOF MS又称为 Mass-Fix，梅奥诊所Rochester分院区自2018年使用MALDI-TOF MS取代免疫固定电泳用于临床检验，除了对M蛋白定量和定性，还可检测M蛋白翻译后修饰。MALDI-TOF敏感性高，检测时间短，与骨髓MFC-MRD 结果的一致性为62％[35]，灵敏度低于克隆特征肽法。QIP-MS为Binding site公司的商业产品，又称为Mass-Fix/QIP-MS，灵敏度为0.005 g/L，费用较MALDI-TOF MS稍高。

MS-MRD是一种快捷、非侵入性的微量M蛋白检查手段，对EMD分泌的微量M蛋白的检出具有一定优势，而对非分泌型的MM无检测优势。由于外周血M蛋白半衰期的存在（IgG约21 d，IgA约10 d），可能导致外周血MS-MRD检测滞后于骨髓MRD。然而，外周血MS-MRD检测与骨髓MRD检测互为补充，二者均阴性的患者生存期更长，在维持治疗期间出现MS-MRD复发的患者PFS欠佳[36]。

3. 外周血循环克隆性浆细胞检测：在肿瘤负荷较低的缓解状态下，外周血循环克隆性浆细胞MRD与骨髓MRD一致性较低，骨髓NGS的阳性率高于外周血，限制了循环克隆性浆细胞检测的应用和推广。一项前瞻性研究显示，骨髓和外周血样本一致性仅为49％，大多数不一致的病例为骨髓MRD 阳性而外周血MRD阴性[37]。

四、MRD的应用与挑战

MRD阴性不仅反映更优的缓解深度，也是有力的预后指标[38]，为MRD作为治疗终点、MRD驱动治疗策略奠定了基础。越来越多的MM初治和复发/难治患者的临床试验将MRD评估纳入相关分析、终点和驱动治疗干预的决定因素。第四届血液和骨髓移植临床试验网络（Blood and Marrow Transplant Clinical Trials Network，BMT CTN）于2020年发布了临床试验MRD检测和报告共识[39]。MRD检测手段的改进和有效结合、如何应用MRD指导临床决策将是未来的研究热点。

目前国际认可的MRD检测为骨髓多参数流式检测和NGS检测，检测敏感性需达到10^−6^，MM MRD 阴性的最低阈值为10^−5^ [39]，MRD阴性阈值<10^−6^水平疗效和预后价值更大。影像学MRD（PET-CT）可作为骨髓MRD的补充。持续MRD低于检测范围更有利于改善预后[40]。根据IMWG的MRD缓解标准，骨髓MRD阴性同时影像学MRD阴性持续1年以上（1年内两次检测）是目前的理想缓解状态[23]。

MRD阴性是目前MM患者最重要的预后因素之一[38]，MRD阴性预示着更长的PFS和OS（与传统定义的CR相比）[41]，特别是在持续MRD阴性的情况下，可以克服基线高危因素所带来的负面预后影响[3]。在临床实践中，国外多个中心利用诱导治疗后MRD状态辅助指导是否进行早期自体造血干细胞移植的临床决策，诱导治疗后MRD阴性的患者可推迟自体造血干细胞移植[42]–[43]。而对于MRD阴性的细胞遗传学高危患者，其生存优于MRD阳性标危患者[43]–[44]。因此，治疗后的MRD状态有助于患者危险分层，也有助于了解不同危险分层患者治疗后耐药模式。MRD动态评估提示疾病复发或进展的患者，可纳入临床研究以探讨MRD驱动治疗的临床获益问题。对维持治疗阶段，是否根据MRD状态调整维持治疗强度和周期，MRD阴性转为阳性是否需要调整治疗，持续MRD阴性的是否能够停药的问题仍有待讨论。目前正在进行的临床试验（NCT04221178、SWOG S1803、GEM2014）将回答维持治疗持续MRD阴性是否可以停药的问题。

另外，MRD是否可作为临床实践和临床试验终点事件也受到广泛关注[3],[45]。已有多项荟萃分析结果支持MRD作为临床试验终点指标[38],[46]。对新药的开发而言，含新药的方案使得MM中位PFS期和OS期长达数年。使用经典的PFS和OS作为主要终点会导致观察周期延长、经费浪费、药物上市延缓、患者用药延迟等弊端。目前正在进行的临床试验EMN20、GMMGHD7、ADVANCE、DREAM-9将聚焦并解答该问题。

MRD的检测时间点和监测频率、如何组合利用不同MRD检测手段以降低检查成本，避免不必要的骨髓采样或PET-CT检查目前尚未达成共识。适合移植的初诊MM患者可以按照治疗阶段的划分进行MRD检测和随访；不适合移植的初诊MM和复发/难治MM患者，由于没有明确的治疗阶段，推荐在指定的周期数或非常好的部分缓解（very good partial response，VGPR）（由于M蛋白半衰期长，患者可在M蛋白未清除前达到骨髓MRD阴性）/CR进行MRD检测和随访。

作为潜在的替代治疗终点，在MRD及其与预后的关系仍待探讨。尽管MRD阴性提示更长的PFS期，仍存在MRD阴性但早期复发的案例[47]。部分缓解后患者处于类意义未明的单克隆丙种球蛋白血症（Monoclonal Gammopathy of Undetermined Significance，MGUS），处于非常惰性的病程长期不复发，MRD对这部分患者的价值有待探讨。对治疗耐受性低的虚弱患者，MRD对治疗和预后的价值有待讨论[48]–[49]。对高危患者，MRD阴性能克服或者改善不良预后，延长生存[3]。那么高危患者接受标准治疗后仍MRD阳性，是否需要调整为更强的方案以达到MRD阴性仍有待讨论。对维持治疗的患者，持续MRD阴性是否可作为停药指征及其与预后的关系有待阐述。维持治疗期间MRD转为阳性，是否需要调整治疗等一系列问题尚待确认[50]–[52]。

新的治疗方法使MM达到了前所未有的缓解深度，MRD将成为未来MM治疗的重要组成部分。不同维度MRD的动态监测有助于临床医师更好地理解疾病发展过程，优化治疗策略，进而改善MM患者预后。
